# The Role of the Lys628 (192) Residue of the Complement Protease, C1s, in Interacting with Peptide and Protein Substrates

**DOI:** 10.3389/fimmu.2014.00444

**Published:** 2014-09-17

**Authors:** Lakshmi Carmel Wijeyewickrema, Renee Charlene Duncan, Robert Neil Pike

**Affiliations:** ^1^Department of Biochemistry and Molecular Biology, Monash University, Melbourne, VIC, Australia

**Keywords:** complement, C1s, C4, substrate, protease, serine protease

## Abstract

The C1s protease of the classical complement pathway propagates the initial activation of this pathway of the system by cleaving and thereby activating the C4 and C2 complement components. This facilitates the formation of the classical pathway C3 convertase (C4bC2a). C1s has a Lys residue located at position 628 (192 in chymotrypsin numbering) of the SP domain that has the potential to partially occlude the S2–S2′ positions of the active site. The 192 residue of serine proteases generally plays an important role in interactions with substrates. We therefore investigated the role of Lys628 (192) in interactions with C4 by altering the Lys residue to either a Gln (found in many other serine proteases) or an Ala residue. The mutant enzymes had altered specificity profiles for a combinatorial peptide substrate library, suggesting that this residue does influence the active site specificity of the protease. Generally, the K628Q mutant had greater activity than wild type enzyme against peptide substrates, while the K628A residue had lowered activity, although this was not always the case. Against peptide substrates containing physiological substrate sequences, the K628Q mutant once again had generally higher activity, but the activity of the wild type and mutant enzymes against a C4 P4–P4′ substrate were similar. Interestingly, alteration of the K628 residue in C1s had a marked effect on the cleavage of C4, reducing cleavage efficiency for both mutants about fivefold. This indicates that this residue plays a different role in cleaving protein versus peptide substrates and that the Lys residue found in the wild type enzyme plays an important role in interacting with the C4 substrate. Understanding the basis of the interaction between C1s and its physiological substrates is likely to lead to insights that can be used to design efficient inhibitors of the enzyme for use in treating diseases caused by inflammation as result of over-activity of the classical complement pathway.

## Introduction

The complement system is of major importance in innate and adaptive immunity (1), but has also been shown to play a major role in several inflammatory diseases (2, 3). Understanding the precise mechanisms whereby the pathways of the system are activated is therefore likely to provide understanding of potential mechanisms to control the system. Once activated following the recognition and initial activation events of the classical pathway, C1s plays a major role in amplifying the initial recognition by cleaving and thus activating the C4 and C2 complement proteins that go on to form the C4bC2a C3 convertase complex (4, 5). It has recently been shown that the activation of the C1s protease is concomitant with the formation of an exosite on the serine protease domain (6, 7) that works in concert with a likely exosite on the CCP domains and the active site to efficiently catalyze the cleavage of C4 to yield the C4b and C4a fragments. In this regard, the interaction between C1s and C4 is likely to have similarity to the MASP-2-C4 interaction, which has been elucidated structurally (8) and biochemically (9–11). Given that the exosite interactions for the classical and lectin pathway proteases are somewhat similar, the focus falls on the active sites of the proteases in order to provide selective inhibitors of the pathways for use in controlling diseases in which they are involved.

**Figure 1:**
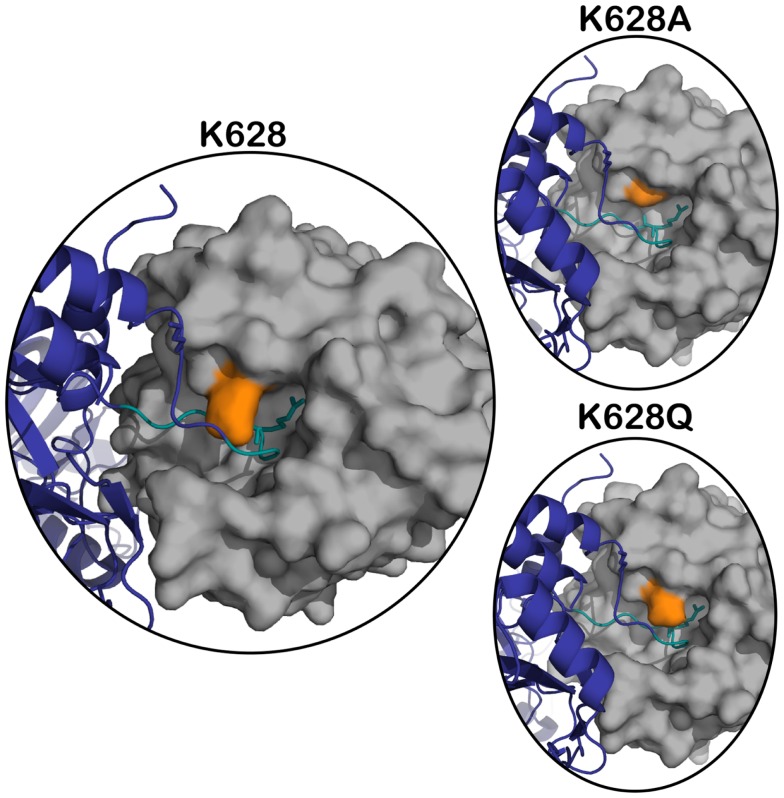
**Superposition of active C1s (gray) (PDB ID: 1ELV) in surface representation, on the structure of the MASP-2 – C4 complex (C4 in navy blue) (PDB ID: 4FXG) in cartoon representation**. The position of K628 (orange) is highlighted with respect to the P4–P4′ cleavage loop of C4 (in teal, P1 arginine sidechain shown in stick). The panels on the right represent the structure in the same orientation with the K628 position mutated to an alanine (top panel) and glutamine (bottom panel).

The X-ray crystal structure of the activated CCP2-SP form of C1s (12) shows that the active site of the enzyme is quite “closed” and restricted from its surrounding environment, which most likely contributes to its high level of substrate specificity (13). During the cleavage of C4, C1s must accommodate a bulky P2 Gln into a relatively small S2 subsite. To date, there is no X-ray crystal structure of C1s with a peptide substrate bound in the active site, and therefore, it is unknown how the protease finds the capacity to accommodate such a large amino acid. The Lys628 (192 in chymotrypsin numbering) is a dominant feature of the active site, as it overlaps the entrance to the crucial S2–S2′ subsites of the enzyme (12) (Figure 1).

The K628 (K192 in chymotrypsin numbering) amino acid has been shown to be vital in many serine proteases. Only 14% of trypsin-like proteases in the human genome contain a lysine at position 192, while 60% contain glutamine and 5.4% glutamate (14). Previous studies in which serine proteases were mutated at position 192 have shown that mutation of this residue can have a variety of effects on the interaction of chymotrypsin-family serine proteases with substrates and inhibitors (14–16).

Here, we have mutated this residue in C1s to Gln and Ala in order to understand its role in the interaction with peptide substrates and C4. The results indicate that the Lys residue at this position in the protease plays a role in facilitating efficient cleavage of C4 in particular.

## Materials and Methods

### Production of recombinant C1s and mutants

Recombinant human C1s and the K628Q and K628A mutants were expressed in *Escherichia coli* as insoluble proteins in the inclusion bodies as described previously (7). The enzymes were denatured and then refolded, following which they were purified by a combination of anion exchange and gel filtration chromatography all as previously described (7). All proteins were obtained in good yield and purity. The zymogen forms of the proteases were activated using immobilized C1r as previously described (7). The activated proteases were titrated using C1-inhibitor (CompTech, TX, USA) to yield the final active concentrations of each enzyme form.

### Cleavage of the REPLi combinatorial substrate library by C1s enzymes

Wild type or mutant C1s forms (400 nM) were tested for their ability to cleave a combinatorial peptide substrate library (REPLi, Mimotopes, Clayton, VIC, Australia) containing 3375 different peptides arranged in 512 pools (17). The assays were conducted in fluorescence assay buffer (FAB) [50 mM Tris–HCl, 150 mM NaCl, 0.2% (w/v) PEG 8000, pH 7.4] at 37°C. Cleavage of the substrates was monitored by measuring the increase in fluorescence intensity from the MeOC fluorophores using 55 s cycles for 30 cycles, with an excitation wavelength of 320 nm and an emission wavelength of 420 nm. The initial velocity of the cleavage was indicated by the slope per unit time of the linear region of the curves.

### Cleavage of peptide substrates containing physiological sequences

Assays were carried out in FAB at 37°C using final substrate concentrations in the range of 0.5–500 μM. The fluorescence quenched C4 P4–P4′ substrate (FQS) [2-aminobenzoate-GLQRALEI-Lys(Dinitrophenol)-NH_2_] and coumarin substrates {C2 P3-P1 substrate [Z-LGR-aminomethylcoumarin (AMC)] and C4 P5-P1 substrate [Z-AGLQR-AMC]} were solubilized in 10% (v/v) dimethylformamide. The rate of increase of fluorescence in the presence of 400 nM C1s (wild type or mutant) was measured on a BMG Technologies FluoStar Galaxy fluorescent plate reader (BMG Labtech, Offenburg, Germany) using an excitation wavelength of 320 nm and an emission wavelength of 420 nm for the FQS and 360/460 nm for coumarin substrates. The initial reaction rate was estimated at a single concentration of enzyme from duplicate measurements over a range of substrate concentrations. In order to determine steady-state reaction constants [*V*_max_ (maximal velocity) and *K*_0.5_ (half saturation constant)], the experimental results were fitted, using the GraphPad Prism Version 5.0 computer program (GraphPad Software, San Diego, CA, USA), to an equation describing positive cooperativity (*V* = *V*_max_[S]h/[S]h + [K_0.5_]h), which defines the relationship between reaction rate (*V*) and substrate concentration ([S]) when more than one binding site applies (18). The catalytic efficiency (*k*_cat_) values were calculated as described previously (18).

### Cleavage of C4

C4 (CompTech, TX, USA) at 1 μM (final concentration) was incubated with increasing concentrations of C1s or mutants (0–5 nM) for 1 h in FAB at 37°C. Reactions were stopped by the addition of reducing SDS-loading buffer and samples were incubated at 90°C for 5 min, then loaded onto 12.5% SDS-PAGE and electrophoresed. Gels were stained with Coomassie blue R-250 stain and destained. The cleavage of the C4 alpha band was analyzed using the gamma band as a loading control. A Typhoon Trio (488, 532, and 632 nm lasers) was utilized for densitometry analysis using the IQTL ImageQuant™ software (1D Gel Analysis) (GE Healthcare, Australia). The densitometry data for the disappearance of the alpha band of C4 was plotted against the log of the concentration of C1s used. The EC_50_ values were derived by fitting the data using non-linear regression to the following equation: *Y* = *Y*_min_ + (*Y*_max_ − *Y*_min_)/{1 + 10^[(LogEC50−X)*h)]^}, where *Y*_min_ is the minimum *Y*-value, *Y*_max_ is the maximum *Y*-value, and *h* is the Hill slope. The analysis was carried out using GraphPad Prism, which output 95% confidence intervals for each value that indicated that the value obtained for the wild type enzyme was significantly different from those obtained for the mutant forms at a 95% confidence level. The experiment was repeated three times with similar results obtained.

The time course of cleavage of C4 (1 μM) by C1s forms at 1 nM was derived by incubating the components for 0, 1, 2, 5, 15, 30, 60, and 120 min, following which the reaction mixtures were treated as described above. The disappearance of the alpha band was quantified by densitometry as specified above.

## Results

**Figure 2:**
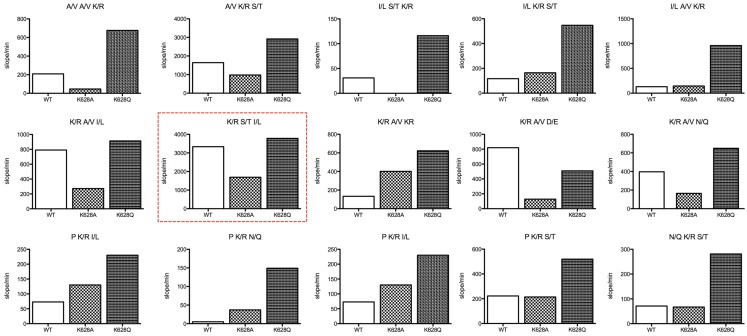
**Bar graphs of the initial velocity values for substrate pools of the REPLi combinatorial peptide substrate library incubated with wild type and K628 mutants of C1s**. The identity of the three amino acid positions varied for each pool displayed is shown above each graph. Initial velocity values for wild type (empty bar), K628A (lighter hatched bar), and K628Q (dark hatched bar) are shown.

The REPLi peptide substrate library provides a rapid means of investigating the substrate specificity of a protease. We have previously characterized the substrate specificity of C1s using a phage display-based analysis (13), which yielded a highly cleaved sequence, YLGR-SYKV, consistent with the preference of this enzyme for a small amino acid, such as Gly, at P2 and a hydrophobic amino acid, such as Leu, at P3. The top ranked substrate for C1s in the REPLi library had the form K/R-S/T-I/L (Figure 2). Since C1s is highly specific for Arg residues at P1 in particular, we can assume this substrate would be cleaved after the K/R position. Since Gly residues occupy all positions other than those being varied in these substrates, this would be the residue found at P2 in this substrate. The strong preference of C1s for P2′ Leu and P1′ Ser residues has also been noted before and the sequence overall is similar to that found at the P1–P2′ of C1-inhibitor, with C4 also having a Leu at P2′.

Overall, the K628Q enzyme mutant was much more active against substrate pools in general than the wild type enzyme, while in general the K628A mutant was less active than the wild type enzyme. This was not true for substrates with a Pro residue at the likely P2 position, which the K628A mutant cleaved better than wild type in general, although less well than the K628Q mutant. The K628A mutant was also more active against the K/R-A/V-K/R pool than wild type. While it cannot be certain where the enzyme would cleave in this substrate, as there are two potential Arg residues that may constitute the P1 residue, there is a likelihood that the second K/R residue would lie within P2′, and thus, it appears that the absence of K628 favors cleavage of this substrate pool. In support of this, the wild type enzyme cleaved the K/R-A/V-D/E pool more rapidly than either mutant. In that case, the D/E residue must sit at P2′ and it appears that the positively charged K628 favors interaction with the oppositely charged D/E residue. Thus, it appears that the K628 residue might indeed be able to interact with the P2′ residues of substrates.

It is worthy of note that the enzyme essentially did not cleave substrates of the form K/R-K/R-I/L (the P1–P2′ sequence of C2), N/Q-K/R-A/V (the P2–P1′ sequence of C4), or I/L-N/Q-K/R (P3–P1 of C4). This indicates that the Lys residue found at the P1′ position in C2 and the Gln residue found at the P2 position of C4 are likely to be prohibitive for cleavage of substrates by C1s. However, the K628Q and K628A mutants of C1s also did not cleave these substrate pools, indicating that K628 does not play a crucial role in preventing interaction with such substrates.

The results obtained with the REPLi peptide substrate library suggested that the K628 residue of C1s plays a role in restricting the substrate specificity of C1s for peptide substrates. In general, the replacement of the Lys residue with the Gln residue found at this position in many other serine proteases increased the cleavage rate of the enzyme for many pools of substrates, while replacement with an Ala had the opposite effect. This general trend was also observed with the Z-LGR-AMC and Z-AGLQR-AMC peptide substrates, representing the P3–P1 and P5–P1 sequences of C2 and C4, respectively (Table 1). The *k*_cat_/*K*_0.5_ value for the K628Q mutant with Z-LGR-AMC was fivefold higher than that for wild type, while the same parameter for K628A was twofold decreased compared to the wild type enzyme. The major determinant of the effects was the change to the *k*_cat_ parameter in each case. The *k*_cat_/*K*_0.5_ for the Z-AGLQR-AMC substrate was increased over 10-fold for the K628Q mutant, while that for the K628A mutant was decreased over 10-fold. Again, the changes to the *k*_cat_ parameter were the major effect noted.

**Table 1:** **Kinetic parameters for cleavage of peptide substrates by wild type C1s and K628 mutants**.

Substrate and parameter		Wild type C1s	C1s K628Q	C1s K628A
Z-LGR-AMC	*K*_0.5_ (μM)	169 ± 7.9	76.2 ± 1.15	212 ± 13.8
	*k*_cat_ (s^−1^)	2.72	6.0	1.77
	*k*_cat_/*K*_0.5_ (M^−1^s^−1^)	1.6 × 10^4^	7.9 × 10^4^	8.3 × 10^3^
Z-AGLQR-AMC	*K*_0.5_ (μM)	245 ± 13.3	65.4 ± 1.91	109 ± 18.9
	*k*_cat_ (s^−1^)	35.33	116.5	1.3
	*k*_cat_/*K*_0.5_ (M^−1^s^−1^)	1.4 × 10^5^	1.8 × 10^6^	1.2 × 10^4^
Abz-GLQRALEI-Lys(Dnp)	*K*_0.5_ (μM)	13.8 ± 0.3	12.3 ± 0.49	12.0 ± 0.6
	*k*_cat_ (s^−1^)	1.14	1.17	1.3
	*k*_cat_/*K*_0.5_ (M^−1^s^−1^)	8.2 × 10^4^	9.5 × 10^4^	1.1 × 10^5^
C4	EC_50_ (nM)	0.16	0.76	0.77

Interestingly, different results were obtained with the Abz-GLQRALEI-Lys(Dnp) fluorescence quenched peptide representing the P4–P4′ sequence of C4. In this case, the *k*_cat_/*K*_0.5_ values for wild type versus the K628Q mutant were essentially the same, while the same parameter for K628A was slightly increased. This indicates that the addition of the prime side residues and the removal of a very bulky coumarin group from the P1′ position of the substrate had major effects on the interaction of the enzymes with the substrate, such that all were essentially equal in their ability to cleave the amino acid sequence found at the cleavage point within the C4 substrate.

**Figure 3:**
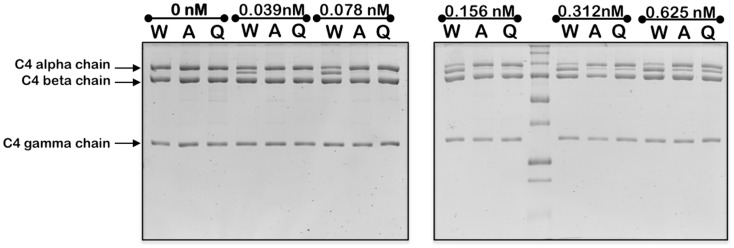
**Cleavage of C4 by increasing concentrations of wild type and mutant forms of C1s**. The cleavage of C4 (1 μM) by the indicated concentrations of wild type (W), K628A (A), and K628Q (Q) forms of C1s was allowed to proceed for 1 h, following which the reaction was stopped by the addition of the loading buffer for SDS-PAGE. The alpha, beta, and gamma chains of C4 are indicated to the left. The cleaved remnant of the alpha chain appears as a band immediately below that of the alpha chain. The experiment was repeated three times with similar results.

**Figure 4:**
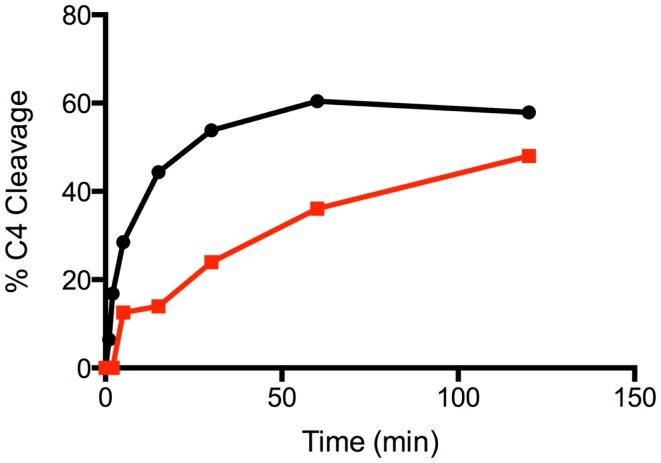
**Time course of the cleavage of C4 by wild type and K628Q forms of C1s**. The cleavage of C4 (1 μM) by 1 nM wild type (black circles) and K628Q (red squares) forms of C1s was allowed to proceed for 0, 1, 2, 5, 15, 30, 60, and 120 min, following which the reaction was stopped by the addition of the loading buffer for SDS-PAGE. Following SDS-PAGE analysis, the disappearance of the alpha chain of C4 was quantified by densitometry and converted into a value indicating the percentage of cleavage.

Mutation of the K628 residue to either a Gln or Ala residue reduced the efficiency of cleavage by approximately fivefold (Table 1). Thus, at concentrations up to 0.3 nM, the wild type enzyme cleaved C4 much more efficiently than either mutant enzyme (Figure 3). The K628Q mutant also cleaved C4 considerably more slowly than the wild type enzyme (Figure 4). This was also found for the K628A mutant (results not shown).

## Discussion

In general, the results obtained with the REPLi library reinforced previous specificity data for this enzyme (13), although it was interesting to note that the I/L-S/T-K/R and I/L-A/V-K/R pools were not well cleaved by the wild type enzyme, somewhat contrary to previous data indicating a preference for Leu residues at the P3 position of substrates for C1s. The mutation of K628 to an Ala residue did not improve the activity of the enzyme against such substrates, but the K628Q mutant cleaved these substrate pools much better, indicating that the mutation to a Gln residue favored such substrates.

The results with the proposed P2′ residues of substrates are similar to what was noted for mast cell chymase, where a K192 residue mediated favorable contacts with negatively charged P2′ residues (19) and thrombin, where E192 restricted the specificity of the enzyme (20). Overall, however, it appears that K628 influences the substrate specificity of C1s at a number of different subsites, indicating that the amino acid sidechain might be interacting with amino acids at a number of different positions within a peptide substrate. This indicates that the residue might be flexible in conformation, as was indicated in the structure of activated C1s previously solved (12).

We have previously shown (7) that the interaction of C1s with C4 can be assessed using analyses that derive the efficiency constant, EC_50_, for cleavage of the substrate by the enzyme. Here, we obtained a somewhat lower EC_50_ for cleavage of the substrate by the wild type enzyme than that reported before [0.16 nM here versus 1 nM reported in Ref. (7)], but nevertheless these efficiency values are within range of each other. The enzymes also were found to cleave C4 more slowly than the wild type enzyme. Our data indicates that although the enzymes could cleave the P4–P4′ sequence of the substrate very similarly, the K628 residue clearly makes an important interaction with the C4 protein substrate that cannot be replicated by the polar Gln residue, suggesting that it is the positive charge of the K628 that is important in this regard. Since cleavage of a substrate that would be expected to bind within the active site was not changed (see Results for C4 P4–P4′ FQS), this would suggest that an interaction between K628 and a substrate residue external to the active site is occurring.

The amino acid found at the 192 position of chymotrypsin-family serine proteases has been shown to be critical in the regulation of interactions with substrates and inhibitors (16, 19, 20). The K628(192) residue of C1s partially occludes the active site by restricting access to the S1 pocket and therefore changes in the interaction with substrates were anticipated. However, it should be noted that in the structure obtained for the active C1s CCP2-SP, K628 is partially disordered, which may suggest the amino acid is quite dynamic in nature (12). It is therefore possible that in the case of the interaction between C1s and C4, the K628 residue is playing a role outside of the active site and interacting with a negatively charged residue of the substrate.

We have previously shown that a positively charged exosite located on the serine protease domain of C1s interacts with a negatively charged site on C4 to yield efficient binding and cleavage of the substrate molecule by the enzyme (7). It is unlikely that it is the same negatively charged site of C4 that is interacting with the Lys628 residue of C1s, but it does suggest that the exosite interactions of the enzyme extend somewhat beyond the originally defined four amino acids of C1s. In the structure of the related MASP-2 enzyme in complex with C4 (8), the Arg residue at the 192 position of this enzyme (630 in numbering of residues in the MASP-2 structure) lies very close to the loop that is cleaved in C4, but it appears to be making little direct contact with the substrate, thus examination of this complex does not provide any obvious clues as to the possible interactions that the K628 residue of C1s might be making. It should be noted that the active site architecture of C1s is markedly different at this position compared to MASP-2, however (12, 21). Modeling of the C1s into the same position as the MASP-2 in this structure (Figure 1) does not provide any further insights, suggesting that the residue may have to change orientation considerably to be able to achieve the new interactions suggested by the data obtained here. In the structure of the zymogen form of C1s (6), this residue faces inwards and away from possible substrate contacts, suggesting that it too is structurally removed from possible substrate interactions, in common with the “main” exosite of the serine protease domain of C1s, which is structurally altered in the zymogen form so that interactions with substrates are minimized in this form of the enzyme.

The studies carried out here give additional insight into the interactions mediated by the K628 residue of C1s. They suggest that in the specific case of the C4 substrate, the residue is playing a role outside of the active site interactions with substrates. However, equally it is clear that the residue can affect the functioning of the enzymes active site in cleaving peptide substrates and thus it must be considered when designing molecules to interact with the active site of the enzyme in order to inhibit it. Such molecules would be expected to have uses in counteracting a number of inflammatory diseases.

## Author Contributions

Lakshmi Carmel Wijeyewickrema helped to design the study, planned and carried out analyses, prepared the figures, and helped to write and revise the manuscript. Renee Charlene Duncan helped to design the study, planned and carried out analyses, prepared the data in publishable format, and helped to write the manuscript. Robert Neil Pike played a major role in designing the study, wrote much of the manuscript, and revised it for final submission.

## Conflict of Interest Statement

The authors declare that the research was conducted in the absence of any commercial or financial relationships that could be construed as a potential conflict of interest.
